# Whether Public Hospital Reform Affects the Hospital Choices of Patients in Urban Areas: New Evidence from Smart Card Data

**DOI:** 10.3390/ijerph18158037

**Published:** 2021-07-29

**Authors:** Fangye Du, Jiaoe Wang, Haitao Jin

**Affiliations:** 1 Key Laboratory of Regional Sustainable Development Modeling, Institute of Geographic Sciences and Natural Resources Research, Chinese Academy of Sciences, 11A, Datun Road, Chaoyang District, Beijing 100101, China; dufy.18b@igsnrr.ac.cn; 2 College of Resources and Environment, University of Chinese Academy of Sciences, Beijing 100049, China; 3 School of Computer, Beijing Information Science and Technology University, Beijing 100101, China; jinht@bistu.edu.cn

**Keywords:** health reform, health-seeking behavior, efficiency of health-seeking, healthcare utilization, big data, Beijing

## Abstract

The effects of public hospital reforms on spatial and temporal patterns of health-seeking behavior have received little attention due to small sample sizes and low spatiotemporal resolution of survey data. Without such information, however, health planners might be unable to adjust interventions in a timely manner, and they devise less-effective interventions. Recently, massive electronic trip records have been widely used to infer people’s health-seeking trips. With health-seeking trips inferred from smart card data, this paper mainly answers two questions: (i) how do public hospital reforms affect the hospital choices of patients? (ii) What are the spatial differences of the effects of public hospital reforms? To achieve these goals, tertiary hospital preferences, hospital bypass, and the efficiency of the health-seeking behaviors of patients, before and after Beijing’s public hospital reform in 2017, were compared. The results demonstrate that the effects of this reform on the hospital choices of patients were spatially different. In subdistricts with (or near) hospitals, the reform exerted the opposite impact on tertiary hospital preference compared with core and periphery areas. However, the reform had no significant effect on the tertiary hospital preference and hospital bypass in subdistricts without (or far away from) hospitals. Regarding the efficiency of the health-seeking behaviors of patients, the reform positively affected patient travel time, time of stay at hospitals, and arrival time. This study presents a time-efficient method to evaluate the effects of the recent public hospital reform in Beijing on a fine scale.

## 1. Introduction

The imbalanced utilization of healthcare services, e.g., overload of top-tier healthcare facilities and underutilization of primary care, remains a prominent problem in public healthcare systems worldwide [1,2]. This imbalanced utilization has been widely recognized as a contributing factor to the waste of medical resources, excessive medical costs, and low health-seeking efficiency [3,4,5]. Therefore, this topic has received great attention from scientists in the field of public health. Understanding the spatiotemporal characteristics of the hospital choices of patients is key to effective interventions toward optimizing the utilization of healthcare services.

In previous studies, survey data have often been used to investigate the utilization of healthcare resources through surveying patient attitudes toward primary care use, healthcare costs, wait times at hospitals, and travel times to hospitals [6,7,8]. However, such data have long been criticized due to the high costs incurred, limited sample representativeness, and low time efficiency [9,10]. Without full consideration of all of the effects of healthcare reform, health planners might be unable to adjust interventions in a timely manner and devise less-effective interventions. Recently, electronic trip records derived from taxis and buses have been used to investigate the spatial and temporal patterns of health-seeking behaviors, as they offer rich information on the movement trajectories of individuals [10,11,12]. For instance, Du et al., (2020) proposed a method to extract transit-based health-seeking trips from smart card data and validate their methods with a health-seeking survey in Beijing, China [13]. Health-seeking trips inferred from electronic trip records offer a large sample size and wide spatial coverage, which may help to better understand the hospital choices of patients more comprehensively. Despite this clear advantage, little attempt has been made to utilize electronic trip records. One explanation might be that transportation records lack information on the purpose of trips, which makes it difficult to identify health-seeking trips.

Public transit is a popular travel mode when accessing healthcare, especially in metropolitan areas. A recent survey on health-seeking behavior in Beijing, China, indicated that about 25% of patients arrive at hospitals by bus [13]. Thus, the present study argues that smart card data have good representativeness for investigating the spatial and temporal patterns of health-seeking behavior. In April 2017, the Beijing became the pilot city to initiate the “Implementation Plan for Comprehensive Reform on Separating Drug Sales from Medical Services”; this will be referred to as Beijing’s public hospital reform in 2017, hereinafter [14]. One of its major goals was to optimize healthcare utilization among tertiary, secondary, and primary hospitals. Using Beijing’s public hospital reform in 2017 as an example, this study assesses the effects of this healthcare reform by comparing the pre-reform hospital choices of patients (April 2015) with their post-reform choices (June 2017), using health-seeking trip data extracted from smart card data. As this study evaluates the effects of public hospital reform with data on real-time awareness of the city, it contributes to the construction of smart cities and the management of these cities [15].

## 2. Literature Review

### 2.1. Hospital Allocation and Health Reforms

Guiding the hospital choices of patients is an efficient way to optimize healthcare utilization and improve healthcare accessibility; therefore, factors associated with the hospital choices of patients have been widely explored. Overall, the factors can be classified as spatial factors (e.g., distance to healthcare facilities), and non-spatial factors (e.g., socio-economic attributes, severity of illness, quality of medical services, medical costs, and healthcare insurance) [16]. Accordingly, a number of measures for guiding the hospital choices of patients have been implemented. Part of the related studies suggested relocating healthcare facilities, especially high-quality healthcare facilities, to rural areas, or the periphery of urban areas, as this measure would improve healthcare accessibility in these areas by shortening the distance to healthcare facilities [10,17,18]. However, core urban areas often have an abundance of healthcare facilities. Thus, the dominant problems of healthcare utilization are not the distance from healthcare facilities, but the imbalanced healthcare utilization among different levels of hospitals, e.g., the overload of tertiary hospitals and the underutilization of primary care facilities. In response to this problem, health reforms were called for in previous practices. Numerous international practices, such as the healthcare reforms of the United Kingdom, Spain, Switzerland, and the Netherlands, demonstrated a gatekeeper as an efficient way to promote the use of primary care [19,20,21]. In China, a new round of healthcare reform has been carried out since 2009. Some of the major tasks of this healthcare reform is to optimize healthcare utilization among different levels of hospitals through a hierarchical medical system and improve the medical quality of primary healthcare [1,22,23]. Specifically, reforms related to guiding the hospital choices of patients and optimizing hospital utilization are summarized in Section 2.2.

### 2.2. Reforms for Optimizing Hospital Utilization in China since 2009

Optimizing hospital utilization is critical toward improving healthcare accessibility and reducing medical costs, which has been called for by health reforms. The last decade of this new round of health reforms in China mainly adopted three measures. The first measure was the improvement of primary medical services. Distrust in healthcare services in primary hospitals is one major reason that impedes the primary healthcare use of patients. To change the attitudes of patients toward primary hospitals, and to promote primary healthcare use, previous reforms have implemented measures to improve the quality of healthcare services in primary hospitals. Prominent examples include the “opinions on deepening healthcare system reform”, issued in 2009 [24], and the “opinions on the pilot of public hospital reform”, issued in 2010 [25]. Second, a number of reforms advocated the realization of differentiated health services at different hospital levels. For example, tertiary hospitals are expected to serve serious and complex illnesses, while secondary and primary hospitals aim to serve patients with chronic illnesses, patients at the recovery stage, and patients with common illnesses. Because of the disparity in service quality between tertiary and primary healthcare facilities, the problems that emerge because of the overload of tertiary hospitals and the underutilization of primary care facilities are very serious. To solve these problems, reforms for building a hierarchical medical system, referral system, and health service consortium have been issued [26,27]. Third, current health reforms address the removal of drug markups [28,29]. The profitability of hospitals in China has relied on drug sales since the 1950s, which might lead to unnecessary drug consumption; thus, increasing medical expenditures of patients. Removing drug markups not only reduces medical expenditures of patients, but also promotes referral between different levels of hospitals and contributes to a balanced healthcare utilization. The specific measures in related reforms are summarized in the Appendix A.

Recent studies have evaluated the effects of public hospital reform by comparing the proportion of patients at each level, of both hospital pre-reform and post-reform, with large-scale statistical data, or by surveying the change of patient attitudes toward primary care use after the reform [30,31]. For instance, Wu et al., (2017) examined public views toward the choice of first-contact care in the hierarchical medical system of China [7]. By focusing on the effects of Beijing’s public hospital reform in 2017, Zhou et al., (2018) found that the number of patients who sought healthcare in secondary and tertiary hospitals decreased significantly, while the number in primary healthcare facilities increased sharply. For their study, Zhou et al., (2018) used data on patient visits at all levels of hospitals from January 2016 to October 2017 [32]. However, little research had focused on the spatial difference of the effects of public hospital reform on the hospital choices of patients.

## 3. Study Design

### 3.1. Case Study—Beijing’s Public Hospital Reform in 2017

In 2012, Beijing began to explore ways to optimize healthcare utilization at different levels at hospitals. After a five-year exploration of pilot hospitals, Beijing took the lead in implementing a public hospital reform, named “Implementation Plan for Comprehensive Reform on Separating Drug Sales from Medical Services”, at all public hospitals, which started on 8 April 2017 [33]. This reform aimed to reconstruct the healthcare service system via the following measures: (i) drug price markups were cancelled in order to reduce the heavy reliance of public hospitals on drug sales; (ii) a medical service fee was implemented to replace drug markups, registration fees, and treatment fees. Charges are tiered by hospital levels (tertiary, secondary, and primary healthcare facilities). Doctors redirect the flow of patients from tertiary hospitals to secondary or primary healthcare facilities. Higher-level hospitals (i.e., tertiary hospitals) and senior physicians (i.e., specialists) can charge higher service fees (Table 1). Third, the reform improved the availability of medicine at primary healthcare facilities, especially for patients with chronic diseases. At primary healthcare facilities, prescription duration for patients with non-communicable diseases was extended from one month to two months.

Beijing was chosen as the pilot city to implement the reform on separating drug sales from medical services. Evaluating the effect of this reform is beneficial as it helps the government to understand the current patterns of health-seeking behavior and helps to smoothen the start of the reform in other cities. Using Beijing’s public hospital reform in 2017 as an example, the present study evaluates the effects of public hospital reform by comparing the spatial and temporal patterns of health-seeking behavior (e.g., tertiary hospital preference, hospital bypass, and the efficiency of the health-seeking behaviors of patients, both pre-reform and post-reform). The specific data collection method and method for inferring health-seeking trips from smart-card data are subsequently introduced.

### 3.2. Data Collection

**Figure 1:**
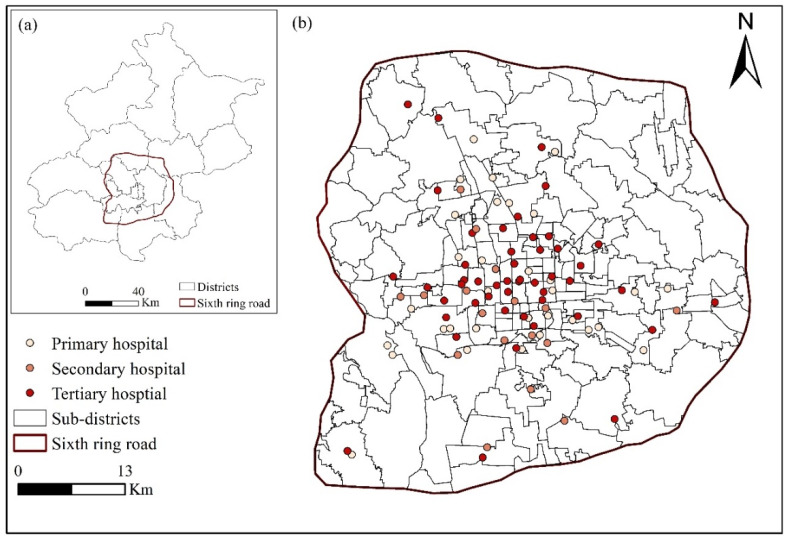
(**a**) Study area within the sixth ring road of Beijing; (**b**) primary, secondary, and tertiary public general hospitals, and subdistricts as basic statistical units.

As public hospital reform is a critical way to optimize healthcare utilization in urban areas, areas within the sixth ring road were selected as the study area (Figure 1a). Datasets regarding hospital facilities and smart-card data within the sixth ring road of Beijing were collected pre-reform (June 2015) and post-reform (April 2017), and are described in the following.

Two categories of healthcare facilities can be found in Beijing, according to their ownership: private hospitals and public hospitals. Public hospitals are fully managed and funded by the state and are commonly characterized by crowding and long wait times. In 2017, 82% of inpatient care was provided by public hospitals [34], and these hospitals are the main targets of the health reform. Thus, a total of 227 general hospitals within the sixth ring road of Beijing were identified from Baidu Map (2015, 2017) [35]. From April 2015 to June 2017, no new general hospital was built, and no current hospital was relocated in the study area. It is worth noting that specialized hospitals, such as dermatology hospital, maternity hospitals, and psychiatric hospitals, were excluded from this analysis because they do not serve the general population. Public general hospitals and private general hospitals were distinguished according to the information on hospital ownership provided by their official websites. Finally, a total of 98 public general hospitals within the sixth ring road of Beijing were extracted, including 32 primary, 17 secondary, and 48 tertiary hospitals (Figure 1b).

Since January 2015, all bus lines in Beijing have adopted a distance-based fare system. This system records trip information of passengers, such as card ID, operation date, boarding stop (latitude and longitude), boarding time, alighting stop (latitude and longitude), and alighting time (Table 2). More than 21 million card records were collected with approximately 6.5 million unique card users between 10 and 12 April 2015, and nearly 30 million smart card records between 5 and 7 June 2017.

### 3.3. Method for Inferring Health-Seeking Trips from Smart Card Data

Patients can be classified into inpatients and outpatients, according to their health-seeking purposes. As inpatients often have severe illnesses, health reform might have no significant effect on their hospital choices. Therefore, this study only focuses on the health-seeking behavior of outpatients (referred to as patients hereafter). Using spatial and temporal constrains proposed by Du et al., (2020) [13], patients’ health-seeking trips were extracted from smart card data, in Beijing, for April 2015 and June 2017. Specifically, the method of inferring health-seeking trips is described in the following. First, based on the unique card ID, the trip chain, including several trip legs, were constructed for each card ID. To identify the origins and destinations of trips, all transfer activities, identified by an activity duration shorter than 20 min, and displacement shorter than 500 m, were identified and removed. For each transfer-free trip chain, trip chains were extracted that contained a pair of alighting and boarding bus stops that were both within walking distance (500 m) of the same health facility. Then, the time duration between arrival trips and departure trips was set within 50 and 300 min. Furthermore, the maximum frequency of health-seeking was set as once every three days, which is consistent with the health-seeking frequency of patients with chronic diseases. As the trip purposes of companions is not to seek healthcare—only one trip in multiple, same, health-seeking trips (regarding boarding and alighting time at all stops, origins, destinations, and time of stay in the healthcare facilities) was utilized.

## 4. Effects of Beijing’s 2017 Public Hospital Reform on Patients’ Hospital Choices

Previous studies often measured the hospital choices of patients by their tertiary hospital preferences and hospital bypass behaviors [36]. In addition, the efficiency of health-seeking behaviors of patients might also be an efficient indicator of their hospital choices, because rational hospital utilization would shorten the patient’s travel time to the hospitals and wait time at the hospital, as well as deconcentrate the arrival time [13,37,38]. The present study investigated the effects of Beijing’s public hospital reform in 2017 on the hospital choices of patients by comparing their patterns in regard to tertiary hospital preferences, hospital bypass, and the efficiency of health-seeking behaviors of patients between pre-reform and post-reform.

### 4.1. Tertiary Hospital Preference

**Figure 2:**
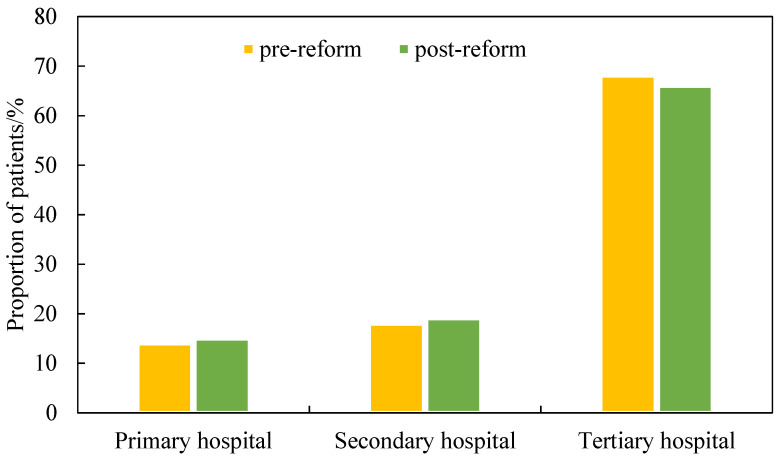
Distributions of patients at different levels of hospitals both pre-reform and post-reform.

Statistically, Beijing’s public hospital reform in 2017 reduced the concentration of patients in hospitals. The Gini coefficient of patient distribution in hospitals decreased from 0.27 in 2015 to 0.24 in 2017. Moreover, the proportion of patients in the top 10% of hospitals decreased by 1.5% post-reform, whereas the proportion in the top 20% of hospitals only decreased by 0.5%. This implies that patient preference for tertiary hospitals decreased. After the reform, the proportion of patients who sought healthcare in tertiary hospitals decreased by 2%, whereas in secondary hospitals and primary hospitals increased (Figure 2). In addition, more than 60% of tertiary hospitals have a decreased proportion of patients. Nevertheless, the proportion of patients in hospitals with famous departments nationwide (such as Tongren Hospital and Anzhen Hospital), did not decrease in response to the reform. This is probably because the specialized treatments in these hospitals could not be replaced by treatments in other hospitals. In general, it could be preliminarily concluded that Beijing’s public hospital reform in 2017 promoted the health-seeking behavior of secondary and primary hospitals and, thus, decreased the pressure on tertiary hospitals.

**Figure 3:**
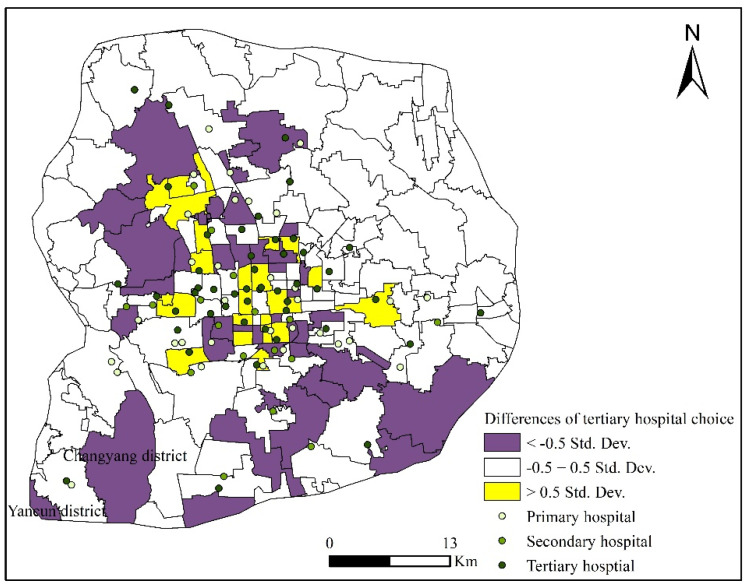
Comparison of the proportion of patients who chose tertiary hospitals pre-reform and post-reform by subdistricts.

Spatially, the pre-reform and post-reform patterns of tertiary hospital choices were compared by computing the difference of the percentage of patients who sought healthcare in tertiary hospitals by subdistricts. This difference is presented in the form of multiples of standard deviations (Figure 3). Multiples close to 0 imply a higher degree of consistency and vice versa. This study assumes that there is no significant difference if the multiples range between −0.5 and 0.5 [13]. After the reform, the preference for tertiary hospitals of patients who lived in subdistricts far from tertiary hospitals decreased. For example, before the reform, patients in the subdistricts Changyang and Yancun were more likely to seek healthcare in Liangxiang Hospital (i.e., a tertiary hospital), whereas the likelihood decreased after the reform. However, for patients near tertiary hospitals, the effects of Beijing’s public hospital reform in 2017 varied by core urban areas and suburban areas. The effect slightly increased the proportion of patients choosing tertiary hospitals in core areas but had no obvious effect on patients in suburban areas. This result is reasonable because the crowdedness of tertiary hospitals in core urban areas decreased after the reform and, thus, more patients were attracted nearby. Tertiary hospitals in suburban areas were not as crowded as those in the core urban areas, even before the reform. Thus, the reform might not change the attitude of patients toward tertiary hospital choices if they live near tertiary hospitals in suburban areas.

### 4.2. Hospital Bypass

Hospital bypass, i.e., the tendency to not seek healthcare at the hospital closest to the patient, is a contributing factor for the inefficient usage of medical resources and one cause of the mentality that “proper healthcare is difficult to get”. This study identifies hospital bypass behavior, if patients do not choose their closest hospital, or hospitals within 3 km of their residence when they seek healthcare [39,40]. The hospital bypass ratio is calculated as the number of patients who showed bypass behavior divided by the total number of patients in a certain area. Statistically, Beijing’s public hospital reform discouraged hospital bypass in the whole study area, as the hospital bypass ratio decreased from 68.1% in 2015 to 65.9% in 2017. Regarding the attributes of hospitals nearby, the bypass ratios were calculated for the patients near primary, secondary, and tertiary hospitals. It is worth noting that patients were regarded as living near high-level hospitals if both low-level and high-level hospitals were located within a 3 km buffer of their residence. The results indicate that patients who lived near primary hospitals had the highest probability of bypassing hospitals, followed by secondary hospitals and tertiary hospitals. For patients who lived close to primary hospitals, the percentage of patients who displayed bypass behavior decreased by about 3% after the reform. Moreover, the percentage of patients who displayed bypass behavior living near secondary hospitals and tertiary hospitals were about 1.5% and 0.3%, respectively. This indicates that Beijing’s public hospital reform exerted the greatest influence on the hospital choice of patients near primary hospitals.

**Figure 4:**
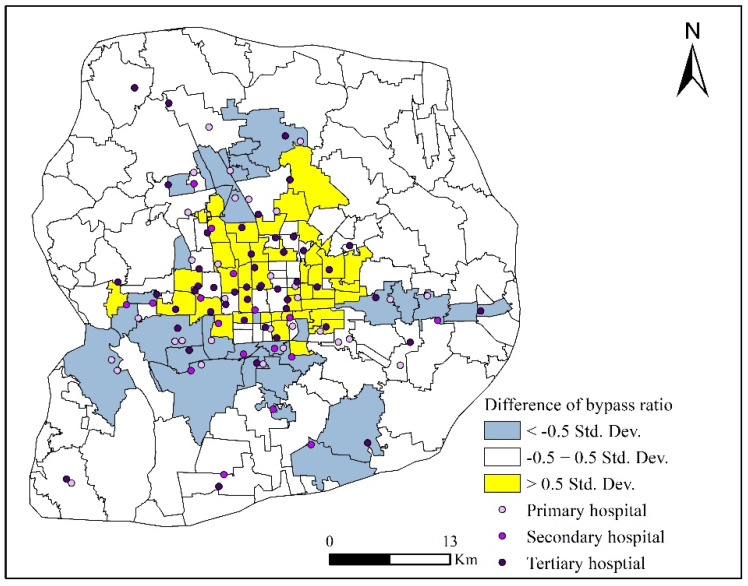
Spatial difference between hospital bypass ratios pre-reform and post-reform.

For comparison and visualization purposes, the spatial distribution of hospital bypass was calculated by subdistricts as the percentage of patients who bypassed nearby hospitals when seeking healthcare. Then, the difference of the hospital bypass ratios between pre-reform and post-reform were computed and mapped in the form of multiples of the standard deviation. As shown in Figure 4, the effects of Beijing’s 2017 public hospital reform on the hospital bypass ratio were closely related to the distributions of healthcare facilities. In subdistricts without healthcare facilities, the reform had no significant effect on the hospital bypass ratio. Patients in these subdistricts had to travel a long distance to seek healthcare both pre-reform and post-reform. However, for subdistricts with healthcare facilities, the reform clearly decreased the hospital bypass ratio in subdistricts located in the suburban areas, whereas the hospital bypass ratio increased in core areas. This is probably because the high medical cost and long travel distance incentivized patients with mild illnesses to seek healthcare nearby. Hence, more healthcare resources are available for patients in core areas, and they could choose facilities more freely.

### 4.3. Efficiency of Patients’ Health-Seeking

In addition to traditional measurements, e.g., travel time to hospitals and wait time, the pattern of arrival time is also a key indicator for the patients’ health-seeking efficiency. This is because patients who seek healthcare in hospitals with a large volume of patients need to arrive early, which represents low health-seeking efficiency (and vice-versa). In addition, the authors argue that the time of stay at a hospital is a better indicator to reflect the efficiency of patients’ health-seeking compared to the wait time. The reason is that the time of stay not only includes the time waiting to see a doctor, but also the time waiting for other medical services, such as blood tests and chest X-rays.

**Figure 5:**
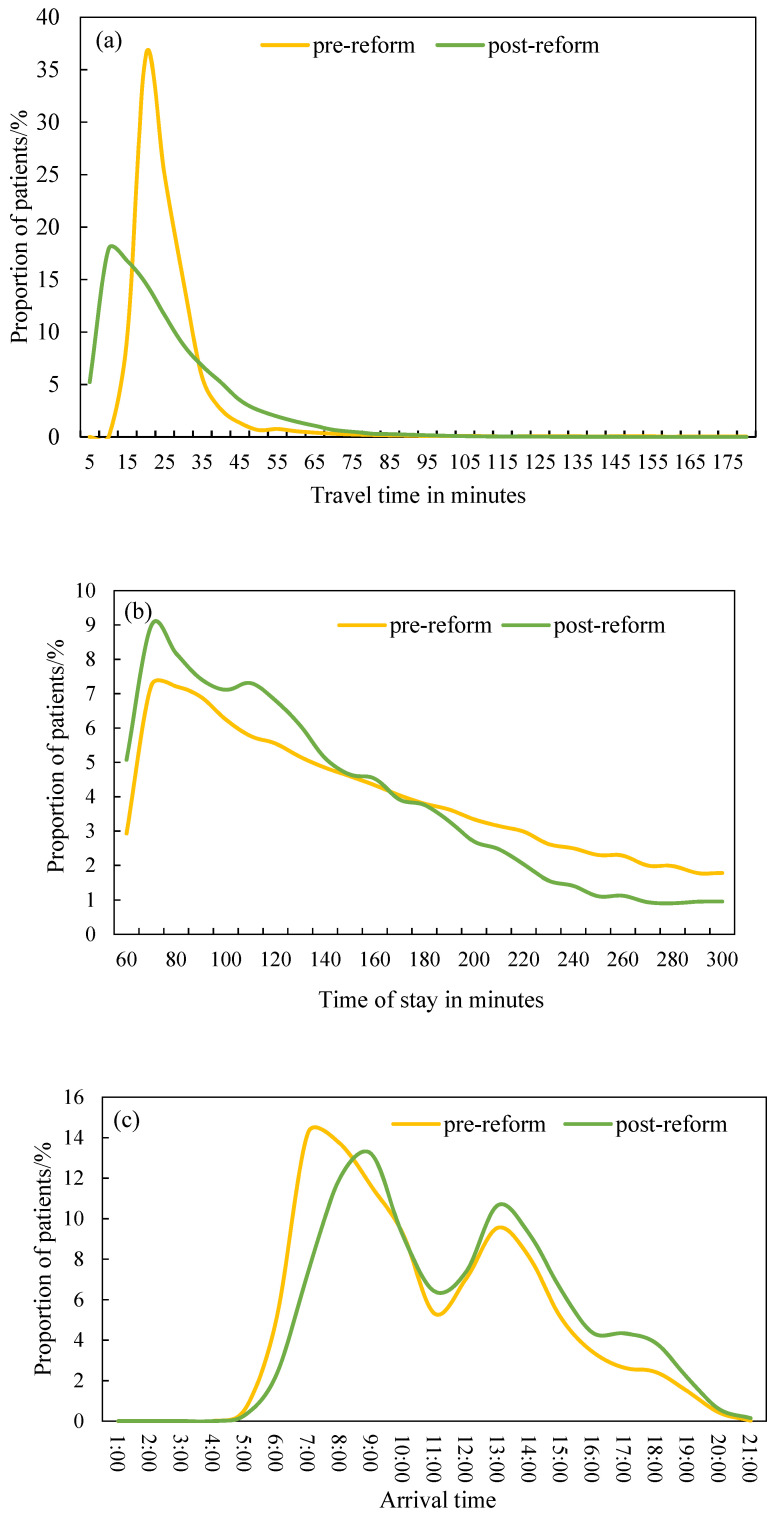
Frequency distributions of travel time to hospitals (**a**), time of stay at hospitals (**b**), and arrival time (**c**) for both pre-reform and post-reform data.

The implementation of Beijing’s public hospital reform in 2017 improved the efficiency of health-seeking by shortening patients’ travel time to hospitals. After the reform, a patient’s average travel time in the study area shortened from 24.3 min to 22.2 min. Moreover, the proportion of patients with travel times less than 30 min increased. For comparison, and visualization purposes, the proportion of patients by time interval was computed as a frequency distribution of travel time. Figure 5a depicts similar frequency distributions of travel time pre-reform and post-reform, both of which show positive skewness distributions, but with different peaks in 20–24 min and 5–14 min. Regarding travel time to different levels of hospitals—Beijing’s public hospital reform exerted an obvious effect on the travel time to tertiary hospitals. The average travel time to tertiary hospitals shortened by 2 min, followed by secondary hospitals (1 min). The reform has not changed patients’ average travel times to primary hospitals.

After the reform, the average time of stay at hospitals shortened from 152.2 to 145.5 min, which is further evidence that the reform enhanced the efficiency health-seeking behaviors of patients. As shown in Figure 5b, the frequency distribution of the time of stay at hospitals pre-reform and post-reform are both unimodal and right-skewed, but the proportions of patients in each time interval are totally different. The proportion of patients with a time of stay at hospitals less than 150 min greatly increased after the reform. A comparison of the effects of Beijing’s public hospital reform in 2017 by hospital level showed that it had the greatest impact on the time of stay at tertiary hospitals. The average time of stay at tertiary hospitals shortened by about 7 min, whereas the time of stay at secondary hospitals and primary hospitals only shortened by about 3 min and 1 min.

A comparison of patients’ arrival time patterns pre-reform and post-reform also supports that Beijing’s public hospital reform improved the efficiency of patients’ health-seeking. Figure 5c shows that both the arrival time pre-reform and post-reform have a similar two-modal distribution, but with different peaks. The arrival time after the reform peaked at 8:00–10:00 a.m. and 1:00–2:00 p.m., whereas the arrival time before the reform peaked at 7:00–9:00 a.m. and 1:00–2:00 p.m. The morning peak hours occurred later. Moreover, the proportion of patients who arrived at the hospitals during the morning peak hours decreased. This is reasonable because Beijing’s public hospital reform in 2017 directed patients with mild illnesses to seek healthcare at low-tier hospitals, which increased the availability of medical resources in top-tier hospitals and reduced the necessity for early arrival. Regarding the different levels of hospitals—the morning peak hours of tertiary hospitals changed from 7:00–9:00 a.m. to 7:00–10:00 a.m. after the reform and, thus, the proportion of patients in each hour decreased. Furthermore, no obvious change of morning peak hours was found in secondary and primary hospitals.

## 5. Discussion

Overall, the results indicate that Beijing’s public hospital reform in 2017 had a positive effect on optimizing hospital choice among different levels of hospital. The main findings can be interpreted as follows:

First, Beijing’s public hospital reform in 2017 helped to promote the utilization of secondary and primary hospitals and share the pressure that tertiary hospitals experienced. Spatially, the effects of the reform on hospital choice and hospital bypass are both closely related to the spatial distribution of hospitals. The reform significantly affected health-seeking behaviors of patients in subdistricts with healthcare facilities. Because of the long travel time and high medical service fees, when seeking healthcare for mild and common illness, patients far away from tertiary hospitals might forgo high-quality medical services and choose nearby healthcare facilities. Thus, as more healthcare resources become available for patients in core areas, they can choose facilities more freely and are more likely to seek high-quality healthcare resources. However, hospital choices by patients in subdistricts without healthcare facilities are not sensitive to the reform. As patients in these subdistricts have to travel long distances when seeking healthcare (both pre-reform and post-reform), the reform might not affect tertiary hospital preference and hospital bypass behavior.

Second, Beijing’s public hospital reform in 2017 shortened both the travel time and the time of stay at hospitals. In addition, the morning health-seeking peak hours were delayed. This is probably because patients with mild illnesses might choose secondary and primary hospitals rather than tertiary hospitals. Moreover, an increased number of patients would not cause pressure for secondary and primary hospitals as the efficiency of patients’ health-seeking in these hospitals retained the pre-reform level. This may also be evidence that the reform promoted the rational utilization of healthcare resources at different levels of hospitals.

Despite this, the effects of Beijing’s public hospital reform in 2017 remain limited, as the distributions of patients at different levels of hospitals only changed slightly. Explanations are summarized in the following. Community hospitals were excluded in this research as they are often close to patients’ homes, and patients are more likely to use non-motorized travel modes when seeking healthcare from community hospitals [41]. After Beijing’s public hospital reform in 2017, a number of patients might prefer seeking healthcare from community healthcare facilities. Therefore, ignoring the data on health-seeking from community healthcare facilities might underestimate the positive effects of the healthcare reform.

To further optimize hospital utilization among different hospital levels, a number of policy implications are proposed, mainly focusing on improving healthcare quality in primary healthcare facilities and strengthening the referral system. First, measures should be taken to establish an effective and reliable primary healthcare system. For instance, primary hospitals should strengthen the medical training of their current doctors and staff. Famous experts could be invited regularly for diagnosis and academic communication. In addition, subsidies for the infrastructure and workforce of primary hospitals can attract more doctors with professional reputations and strong skills. Second, the establishment of the “Health Services Consortium” should be accelerated in Beijing, as this consortium aims to strengthen the share of patients and resources among tertiary hospitals, secondary hospitals, and primary hospitals. This strengthening is an efficient way to promote the hierarchical medical treatment and optimize the usage of different hospital levels. Third, a differential reimbursement system should be implemented, offering payment incentives for services delivered at primary hospitals. In addition, the reimbursement ratio of patients who go to high-level hospitals without a referral should be reduced or removed entirely. Of course, patients with a history of severe illness may be treated differently. Finally, a mandatory gatekeeper could also be considered.

Despite all of these advantages, two limitations need to be discussed. First, this study simply defined hospital bypass as patients who chose not to use their closest hospital or hospitals within 3 km of their residence when seeking healthcare, which does not consider the designated hospitals of patients. For example, the designated hospitals of workers are often decided by their employers, which might not be close to their locations of residence, as identified from smart card data (this difference originates from differences between the locations of residence and workplace). Other workers may also choose a tertiary hospital as their designated hospital, which might be a famous hospital, it might also be far away from their locations of residence or workplace. These phenomena might contribute to a high hospital bypass ratio. Second, the reliability of these results remains debatable because of the small data problem and, thus, further interpretations should focus on subdistricts with a small number of patients.

## 6. Conclusions

Due to increased standards of living, patient preferences for high-quality resources are becoming increasingly obvious, although this exacerbates the overcrowding of top-tier hospitals and underutilization of primary hospitals. Numerous healthcare reforms have been carried out at various governmental levels in China to alleviate these problems. To evaluate the effectiveness of public hospital reforms, a series of studies assessed whether the implemented public hospital reforms influenced patient attitudes toward primary care and assessed related factors via traditional survey data. However, relatively little research efforts have been directed toward the effects of a public hospital reform on the spatial and temporal patterns of health-seeking behavior. Therefore, little is known about the effects of public hospital reforms on the spatial differences of the hospital choices of patients. Without such knowledge, health planners might be unable to devise effective interventions. One major contribution of this study is the presentation of a comprehensive perspective of the effects of public hospital reform on the hospital choices of patients and associated spatial differences. This study used a large-volume dataset with high spatiotemporal resolution, instead of simply surveying attitudes of patients toward primary care use. As public hospital reform in China is still being implemented, this research provides a time-efficient and comprehensive way to assess the effectiveness of public hospital reform. As Beijing is the first city to implement the reform of separating drug sales and medical services across the whole city—the case study on Beijing may also serve as a reference for related practices in other cities nationwide.

## Figures and Tables

**Table 1:** Registration fee (RF) pre-reform and medical service fee (MSF) post-reform. Unit: CNY.

	Tertiary Hospital		Secondary Hospital		Primary Hospital	
	RF	MSF	RF	MSF	RF	MSF
Junior physicians	5	50	5	30	5	20
Associate chief physician	7	60	7	50	7	40
Chief physician	9	80	9	70	9	60
Senior physicians	14	100	14	90	14	80

**Table 2:** Examples of smart card data records.

Card ID	Trip Leg ID	Operating Date	Boarding Stop			Alighting Stop		
			Latitude	Longitude	Time	Latitude	Longitude	Time
0101	1	11 April 2015	116.402	39.942	07:00:00	116.453	39.956	07:36:00
0101	2	11 April 2015	116.453	39.956	09:08:00	116.468	39.952	10:12:00
0101	3	11 April 2015	116:468	39.952	17:25:10	116.402	39.942	18:10:20
0012	1	6 June 2017	116.470	39.867	8:10:00	116.450	39.856	8:30:20
0012	2	6 June 2017	116.450	39.856	12:30:43	116.470	39.867	12:46:53
0050	1	7 June 2017	116.398	39.975	11:30:35	116.444	39.711	12:30:40

## Appendix A

**Table A1:** Reforms on medical services and drug sales since 2009.

Reform	Year	Measures on Medical Services	Measures on Drug Supply
Opinions on deepening the healthcare system reform.	2009	Completed the primary healthcare system and constructed primary healthcare facilities.	Introduced production, circulation, price, usage, and reimbursement policies for essential drugs.
Opinions on the pilot of public hospital reform.	2010	Completed the plan of the construction of primary healthcare facilities and implemented public hospital reform in pilot cities.	Implemented an essential drugs system in 60% of primary healthcare facilities and prioritized essential drugs in other healthcare facilities.
Opinions on the pilot of public hospital reform in urban areas.	2015	Built a compensation mechanism for reduced drug markups and implemented a relevant reform in pilot cities.	Removed drug price addition and reduced price of medical investigations.
Guiding opinions of the general office of the state council on pushing forward the formation of a hierarchical medical system.	2015	Differentiated the functions at different levels of hospital, enhanced the primary healthcare services, and promoted IT application in the medical system.	--
Notice on promoting the pilot of a hierarchical medical system.	2016	Selected 266 cities as pilot cities to implement a hierarchical medical system, enhanced the primary healthcare services, established a family doctor system, and built a health service consortium.	--
Notice on the implementation of a comprehensive public hospital reform.	2017	All cities should have issued a plan of comprehensive public hospital reform before 31 July 2017, and implemented it until 30 September 2017.	Removed drug markups (except Chinese Herbal Medicines) in all public hospitals until 30 September 2017.
